# Down-Regulation of miR-101 in Endothelial Cells Promotes Blood Vessel Formation through Reduced Repression of EZH2

**DOI:** 10.1371/journal.pone.0016282

**Published:** 2011-01-28

**Authors:** Michiel Smits, Shahryar E. Mir, R. Jonas A. Nilsson, Petra M. van der Stoop, Johanna M. Niers, Victor E. Marquez, Jacqueline Cloos, Xandra O. Breakefield, Anna M. Krichevsky, David P. Noske, Bakhos A. Tannous, Thomas Würdinger

**Affiliations:** 1 Neuro-oncology Research Group, Departments of Neurosurgery and Pediatric Oncology/Hematology, Cancer Center Amsterdam, VU University Medical Center, Amsterdam, The Netherlands; 2 Molecular Neurogenetics Unit, Departments of Neurology and Radiology, Massachusetts General Hospital, and Neuroscience Program, Harvard Medical School, Boston, Massachusetts, United States of America; 3 Center for Cancer Research, National Cancer Institute at Frederick (NCI-Frederick), Frederick, Maryland, United States of America; 4 Department of Neurology, Harvard Medical School, Brigham and Women's Hospital, Boston, Massachusetts, United States of America; CNRS, France

## Abstract

Angiogenesis is a balanced process controlled by pro- and anti-angiogenic molecules of which the regulation is not fully understood. Besides classical gene regulation, miRNAs have emerged as post-transcriptional regulators of angiogenesis. Furthermore, epigenetic changes caused by histone-modifying enzymes were shown to modulate angiogenesis as well. However, a possible interplay between miRNAs and histone-modulating enzymes during angiogenesis has not been described. Here we show that VEGF-mediated down-regulation of miR-101 caused pro-angiogenic effects. We found that the pro-angiogenic effects are partly mediated through reduced repression by miR-101 of the histone-methyltransferase EZH2, a member of the Polycomb group family, thereby increasing methylation of histone H3 at lysine 27 and transcriptome alterations. *In vitro*, the sprouting and migratory properties of primary endothelial cell cultures were reduced by inhibiting EZH2 through up-regulation of miR-101, siRNA-mediated knockdown of EZH2, or treatment with 3-Deazaneplanocin-A (DZNep), a small molecule inhibitor of EZH2 methyltransferase activity. In addition, we found that systemic DZNep administration reduced the number of blood vessels in a subcutaneous glioblastoma mouse model, without showing adverse toxicities. Altogether, by identifying a pro-angiogenic VEGF/miR-101/EZH2 axis in endothelial cells we provide evidence for a functional link between growth factor-mediated signaling, post-transcriptional silencing, and histone-methylation in the angiogenesis process. Inhibition of EZH2 may prove therapeutic in diseases in which aberrant vascularization plays a role.

## Introduction

Angiogenesis - the formation of new blood vessels - occurs during tissue growth and development, but also during wound healing and cancer [1], [2]. Angiogenesis is a balanced process controlled by pro- and anti-angiogenic molecules [3]. VEGF has been identified as one of the most potent factors involved in angiogenesis [4]–[6]. VEGF, produced in large amounts by cancer cells during tumor growth, interacts with its receptors VEGFR1 and VEGFR2 thereby causing endothelial cell survival, proliferation, and sprouting [7].

Recently, it was shown that angiogenesis is also controlled by miRNAs [8], [9]. miRNAs comprise a large group of endogenous non-coding RNAs that can block mRNA translation or negatively regulate mRNA stability and thereby play a central role in regulating gene expression [10], [11]. We previously showed that VEGF signaling in primary endothelial cell cultures caused overexpression of VEGFR2 in a positive feed-forward loop, which is at least partly regulated by loss of miRNA-mediated control of VEGF receptor degradation. Moreover, we found that glioblastoma cells, notorious for their VEGF production, elicited a similar response when co-cultured with endothelial cells [11].

Another class of potential regulators of gene expression is the group of chromatin modulators involved in histone modification, such as histone deacetylases [12], [13] and histone methyltransferases [14]. Polycomb group proteins (PcG) function as transcriptional repressors that silence specific sets of genes through chromatin modification [14]. PcG proteins act together in polycomb repressive complexes (PRC). PRC2 includes enhancer of zeste 2 (EZH2), suppressor of zeste 12 (SUZ12), and embryonic ectoderm development (EED). EZH2 is the catalytically active component of PRC2 and is capable of trimethylating histone H3 at lysine 27 (H3K27me3) [15]–[20]. Expression profiling indicated that EZH2 transcripts are up-regulated in ovarian carcinoma-associated endothelial cells [21], and modulated EZH2 expression was described to affect genes associated with endothelial differentiation [22], [23]. It was also described that EZH2 expression is regulated by miRNAs including miR-26 [24], miR-214 [25], and miR-101 [24], [26]–[28].

Here we report that EZH2 is up-regulated in angiogenic endothelial cells and that miR-101 is down-regulated in primary endothelial cells exposed to VEGF or glioblastoma cells, as well as in blood vessel endothelial cells that were isolated from vascularized glioblastomas from patient samples. We confirm that EZH2 is a direct post-transcriptional target of miR-101 and that VEGF-mediated down-regulation of miR-101 results in increased expression of the histone methyltransferase EZH2 in angiogenic endothelial cells. Finally, we show that inhibition of EZH2 histone methyltransferase activity inhibits vascularization in glioblastomas *in vivo*, suggesting a possible therapeutic potential for EZH2 inhibitors in the many diseases in which aberrant vacularization plays a role.

## Results

### miR-101 directly targets EZH2 and is down-regulated in tumor-associated endothelial cells

**Figure 1:**
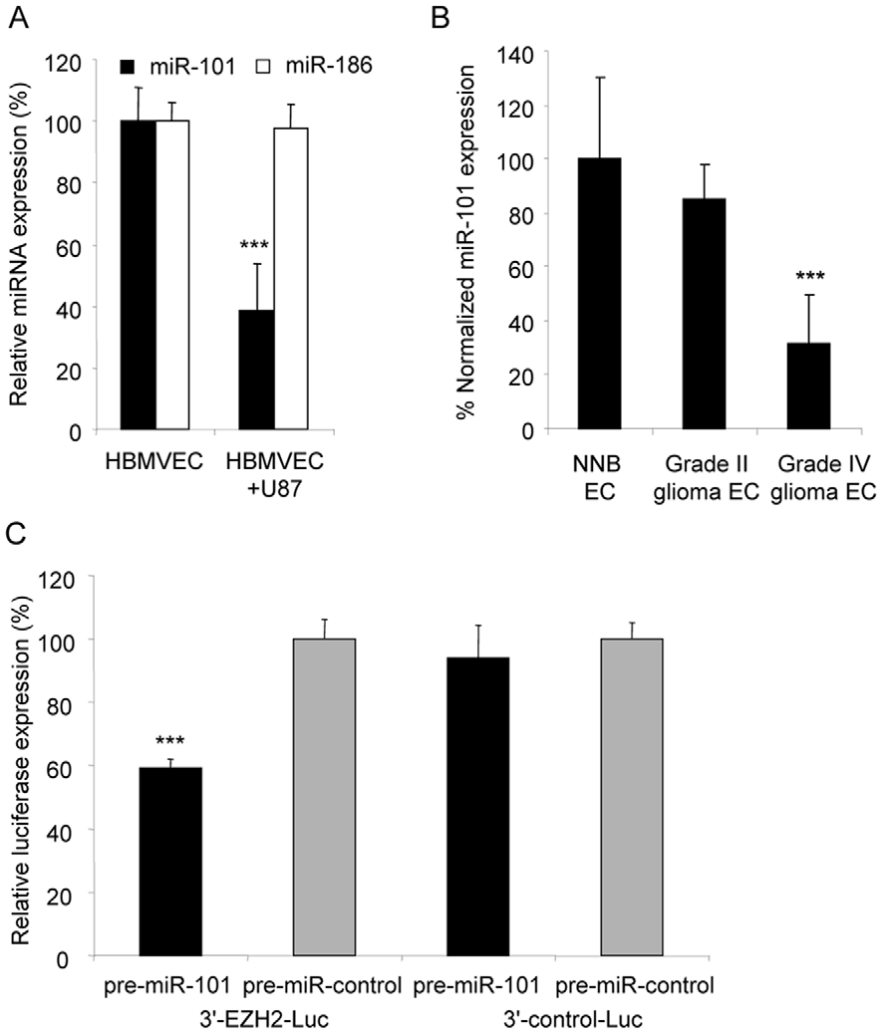
miR-101 is down-regulated in angiogenic endothelial cells and directly targets EZH2. (A) Down-regulation of miR-101 in HBMVECs exposed to U87-GFP glioblastoma cells confirmed by qRT-PCR analysis. RNA extracted from CD31+ HBMVECs cultured in the presence or absence of U87 glioblastoma cells was analysed by qRT-PCR with primers/probes specific for miR-101 and miR-186. The data were normalized to the levels of GAPDH mRNA in each sample. (B) RNA extracted from individual glioma endothelial samples was analyzed by qRT-PCR for expression levels of miR-101 and miR-186. All values were normalized to GAPDH mRNA levels in the same samples. (C) Relative expression of firefly luciferase activity as determined by reporter assay in 293T cells. Luciferase expression was determined 24 h after co-transfection of miR-101 precursor or control miRNA. Luciferase activity of the EZH2 3′UTR reporter vector in the miR-101 transfected cells was significantly reduced as compared to the controls. (n = 3) Error bars indicate s.d. *p<0.05, ***p<0.001 by t test.

We recently showed that various miRNAs are deregulated in human brain microvascular endothelial cells (HBMVECs) co-cultured with U87 glioblastoma cells [11]. Here, a ∼3-fold down-regulation of miR-101 was confirmed by qRT-PCR analysis using miR-186 and GAPDH as normalization controls, both of which were uniformly expressed in endothelial cells in the presence or absence of tumor cells (Fig. 1A). In addition, we examined the expression of miR-101 in angiogenic endothelial cells derived from gliomas resected from patients. Hereto, endothelial cells were isolated from blood vessels derived from three glioblastomas and three grade II gliomas, as well as from non-neoplastic brain [29]. All tumor samples were highly angiogenic as confirmed by CD31 immunohistochemical analysis. We observed reduced miR-101 expression levels in glioma blood vessels as compared to normal brain vessels, as determined by qRT-PCR analysis (Fig. 1B). We were particularly interested in miR-101 since this miRNA was recently shown to interact with EZH2 in different types of cancer [26], [27], and predicted to bind the EZH2 3′-UTR at two sites [30]. To confirm that miR-101 regulates the 3′-UTR of EZH2, we transfected a firefly luciferase (Fluc) reporter vector encoding the wild type 3′-UTR of EZH2 or lacking the EZH2 3′-UTR (control) into 293T cells. Overexpression of miR-101 by pre-miR-101, but not by pre-miR-control, resulted in a decrease in Fluc expression in the cells expressing the reporter with the 3′-UTR of EZH2. No effects were observed using the control vectors lacking the EZH2 3′-UTR (Fig. 1C).

### EZH2 is up-regulated in tumor-associated endothelial cells

**Figure 2:**
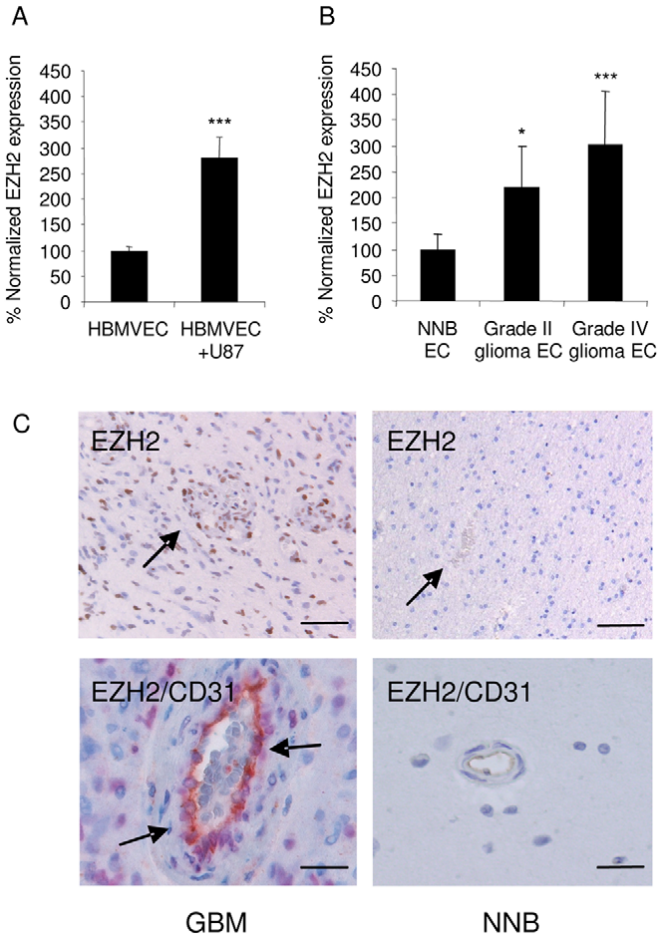
EZH2 is up-regulated in angiogenic endothelial cells and is controlled by miR-101. (A) Overexpression of EZH2 in HBMVECs exposed to U87-GFP glioblastoma cells confirmed by qRT-PCR analysis. RNA extracted from CD31+ HBMVECs cultured in the presence or absence of U87 glioblastoma cells was analysed by qRT-PCR with primers specific for EZH2. The data were normalized to the levels of GAPDH mRNA in each sample. (B) RNA extracted from individual glioma endothelial samples was analyzed by qRT-PCR for expression levels of EZH2. All values were normalized to GAPDH mRNA levels in the same samples. (n = 3) Error bars indicate s.d. *p<0.05, ***p<0.001 by t test. (C) Immunohistochemical staining for EZH2, or EZH2 and CD31, in glioblastoma blood vessels. While glioblastoma sections show positive nuclear staining for EZH2 in tumor blood vessels, staining is absent in NNB. In the top panels EZH2 stains brown and arrows indicate blood vessels. In the bottom panels EZH2 stains purple and CD31 stain brown. Scale bar  = 50 µm (top panels) or 15 µm (bottom panels).

To determine whether EZH2 is overexpressed in angiogenic endothelial cells we analyzed EZH2 mRNA and protein expression. We found the expression of EZH2 mRNA to be increased in HBMVECs after co-culture with human U87 glioblastoma cells (Fig. 2A). In addition, we analyzed EZH2 mRNA expression in glioblastoma blood vessel endothelial cells resected from patient samples as described above. qRT-PCR revealed that EZH2 expression levels were up-regulated in glioblastoma blood vessels as compared to normal brain vessels (Fig. 2B). The overexpression of EZH2 mRNA in the glioma-associated endothelial cells was in line with previously described mRNA expression data of ovarian carcinoma-associated endothelial cells [21]. Upon examination of the EZH2 immunohistochemistry of vascularized glioblastoma and non-neoplastic brain tissue slices, we noticed positive nuclear staining for EZH2 in the endothelial cells of newly formed blood vessels in the glioblastoma samples, but not in the non-neoplastic brain tissues (Fig. 2C). These results indicate an inverse correlation of miR-101 and EZH2 expression levels in angiogenic endothelial cells and led us to further investigate the role of deregulated EZH2 expression during angiogenesis.

### VEGF down-regulates miR-101 expression in endothelial cells resulting in EZH2 up-regulation

**Figure 3:**
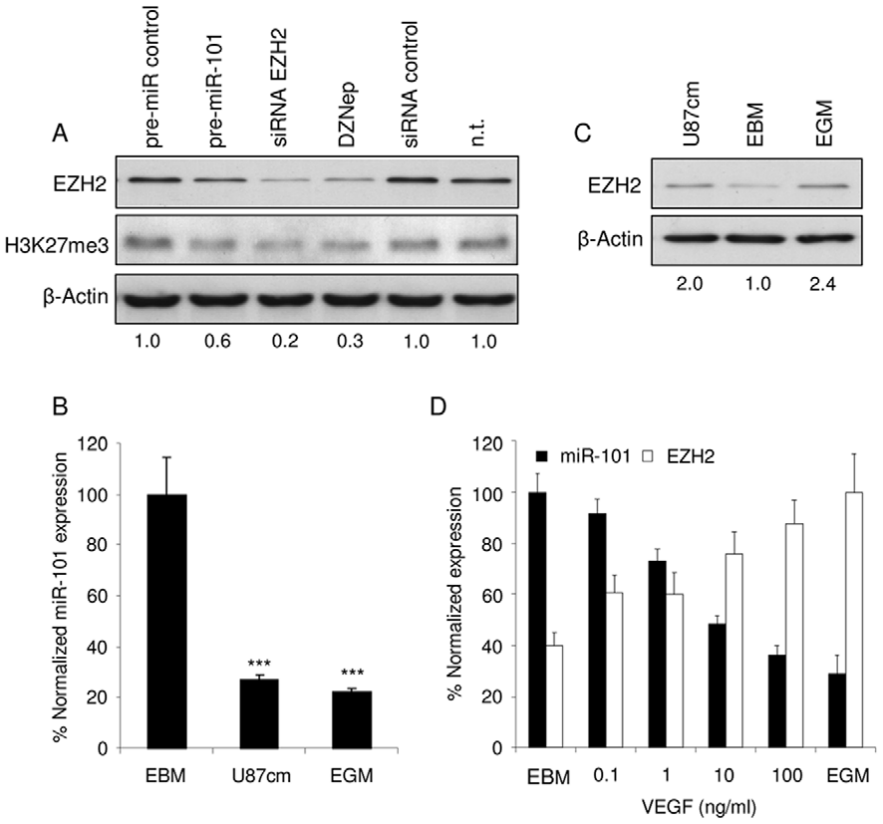
VEGF down-regulates miR-101 expression in endothelial cells resulting in EZH2 up-regulation. (A) HBMVECs protein expression analysis of EZH2 and H3K27me3 following transfection of HBMVECs with pre-miR-101, EZH2 siRNA, non-related control molecules or treatment with DZNep. Numbers indicate relative EZH2 protein expression normalized against β-Actin expression. (B) Inhibition of miR-101 in HBMVECs exposed to U87 secreted factors was confirmed by qRT-PCR analysis. RNA extracted from HBMVECs cultured in EBM, EGM or EBM derived from U87 glioblastoma cells was analyzed by qRT-PCR with primers specific for miR-101. The data were normalized to the levels of GAPDH mRNA in each sample. (C) HBMVECs protein expression analysis of EZH2 following culturing in either EBM, EGM or EBM derived from U87 glioblastoma cells. Numbers indicate the relative expression of EZH2 compared to cells cultured in EBM. (D) HBMVECs were grown in EBM supplemented with different amounts of VEGF for 24 h, after which RNA was isolated to determine the expression levels of miR-101and EZH2 by qRT-PCR. The data were normalized to the level of GAPDH mRNA in each sample. (n = 3) Error bars indicate s.d. *p<0.05, ***p<0.001, t test.

To determine whether EZH2 expression in HBMVECs could be suppressed by up-regulation of miR-101 we performed Western blot analysis for EZH2 after transfection of cells with pre-miR-101, siRNA directed against EZH2, or non-related control oligonucleotides. We also included lysates from cells treated with the S-adenosylhomocysteine hydrolase inhibitor DZNep, a potent inhibitor of EZH2 histone methyltransferase activity [31], [32]. Treatment of the endothelial cells with DZNep, EZH2 siRNA, or pre-miR-101, all markedly reduced EZH2 protein levels. EZH2 gene silencing also decreased histone 3 methylation at lysine 27, as determined by Western blot (Fig. 3A). To establish whether tumor-derived soluble factors are sufficient to induce differential expression of miR-101 and EZH2, HBMVEC cells were cultured in endothelial basal medium (EBM), EBM supplemented with an angiogenic cocktail (EGM), or EBM conditioned culture medium derived from U87 glioblastoma cells (U87cm). We used qRT-PCR to show down-regulation of miR-101 levels in HBMVECs cultured in U87cm or EGM (Fig. 3B). Interestingly, we were not able to measure significant and reproducible miR-101 down-regulation in HUVECs upon exposure to U87 conditioned medium, in contrast to HBMVECs (Fig. S1A). In parallel to the miR-101 down-regulation, EZH2 protein levels were up-regulated in HBMVECs grown in either U87cm or EGM (Fig. 3C) as well as in HUVECs grown in EGM but not reproducibly in HUVECs grown in U87cm (Fig. S1B). EGM and U87cm both contain many growth factors. VEGF in particular, appears to play a major role in angiogenesis [11], [33]. To address the role of VEGF in regulating miR-101 and EZH2 expression, HBMVECs were cultured for 24 h in EBM supplemented with different amounts of VEGF, or in EGM, after which total RNA was isolated and miR-101 and EZH2 expression levels were determined by qRT-PCR (Fig. 3D). An amount of 1 ng/ml of VEGF was sufficient to significantly down-regulate miR-101 levels in HBMVECs and at higher doses its inhibitory effect almost equalled that of the angiogenic cocktail used in EGM medium, indicating that VEGF plays an important role in miR-101 regulation in endothelial cells. In parallel to miR-101 down regulation, EZH2 levels were significantly increased after VEGF stimulation of HBMVECs.

### mRNA profiling of VEGF and EZH2 modulated endothelial cells

**Figure 4:**
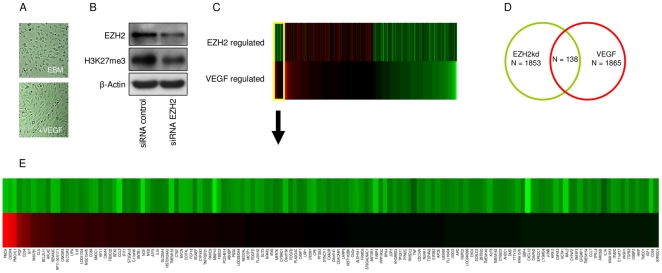
mRNA profiling of VEGF and EZH2 modulated endothelial cells. (A) Primary human brain microvascular endothelial cells (HBMVECs) were cultured in the absence (top) or presence (bottom) of VEGF. (B) Western blot analysis of EZH2 and H3K27me3 expression in HBMVECs upon silencing of EZH2 by EZH2 siRNA. (C) Heatmap representation of top 5% percentile genes up-regulated by EZH2 knock down or down-regulated by VEGF stimulus, the overlapping section of genes regulated by both EZH2 knock down and VEGF stimulus is shown on the left. The top bar represents Log2 fold change ratios of EZH2kd (all >0), the bottom bar represents Log2 fold change ratios of VEGF stimulus (all<0). (D) Venn diagram showing a significant overlap of EZH2 regulated and VEGF regulated genes. Top 5% percentile genes up-regulated by EZH2 knock down or top 5% percentile genes down-regulated by VEGF stimulus were considered potential targets. (E) Detail of heatmap, showing the cluster of genes that are regulated by both EZH2 knock down and VEGF stimulus.

To gain insights into target genes affected by EZH2 mediated gene silencing in the presence of VEGF stimulation, we suppressed EZH2 in HBMVECs cultured in the presence of VEGF and subsequently isolated the RNA. We then used Agilent 44K gene expression arrays to determine mRNA profiles and identify genes whose expression levels were changed by EZH2 silencing in the presence of VEGF. Simultaneously, we used expression arrays to identify a pro-angiogenic gene profile in HBMVECs stimulated by VEGF. VEGF stimulation significantly induced elongation of the endothelial cells (Fig. 4A) indicating angiogenic activation [34]. EZH2 knockdown efficiency and H3K27me3 reduction in the array samples were confirmed by Western blot analysis (Fig. 4B). To identify which VEGF target genes are potentially depending on EZH2 regulation a comparison was made between the top 5 percentile genes whose expression was increased after EZH2 silencing (n = 1865) and the top 5 percentile genes down-regulated by VEGF stimulation (n = 1853) (Fig. 4C, D). We identified a significant overlap of 138 genes (Fig. 4E) in both sets (p<10-9). Of note, the recently reported EZH2 target gene VASH1 [35] was also identified in the set of genes both regulated by VEGF and EZH2.

### Inhibition of EZH2 reduces endothelial tubule formation *in vitro*

**Figure 5:**
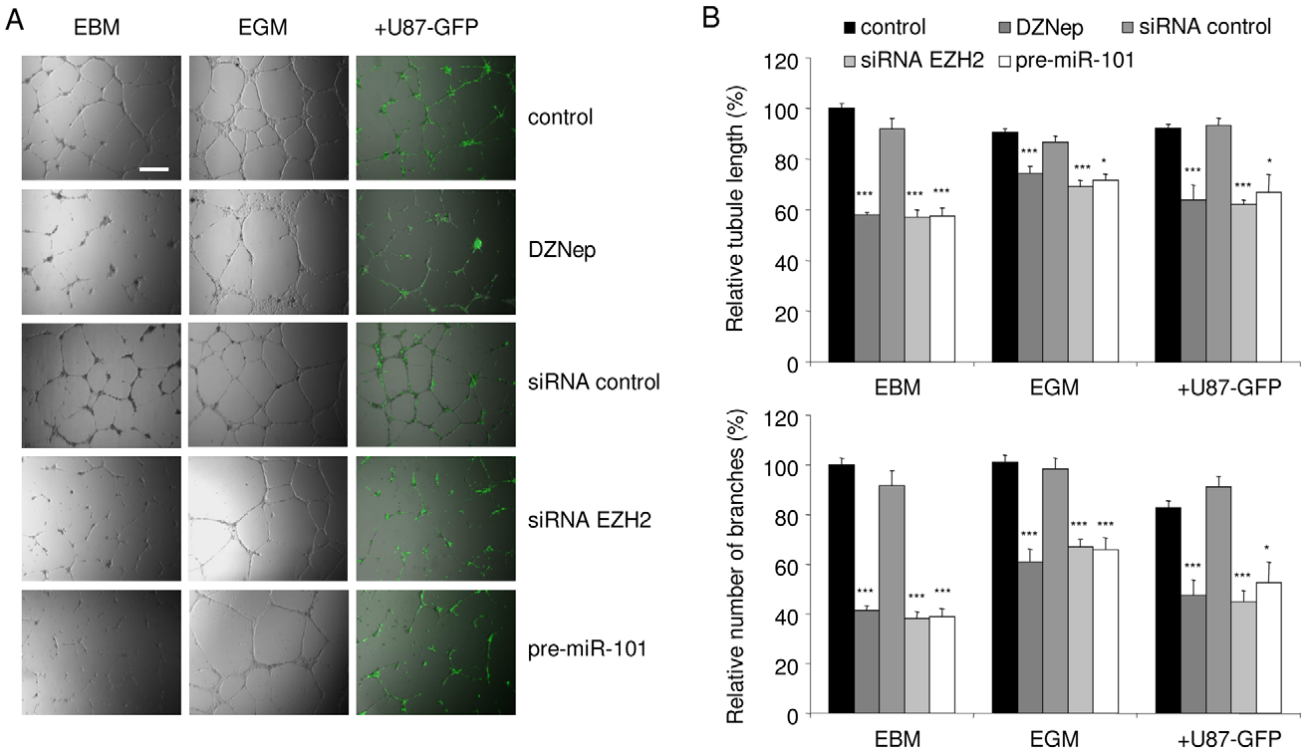
Inhibition of EZH2 reduces endothelial tubule formation *in vitro*. (A) HBMVECs were cultured on Matrigel coated plates in EBM, EGM, or EBM supplemented with U87-GFP cells. Scale bar  = 450 µm. Inhibition of EZH2 in HBMVECs, either by transfection with pre-miR-101, EZH2 siRNA, or treatment with DZNep significantly reduced tubule formation as compared to control cells. Tubule formation was assessed as tubule length and branching. (B) Quantitation of tubule length and branching using ImageJ software. (n = 3) Error bars indicate s.d. *p<0.05, ***p<0.001, t test.

To further assess the function of EZH2 up-regulation in angiogenesis, we examined EZH2 inhibition in angiogenic HBMVECs in culture. HBMVECs were cultured in EBM, EGM, or were co-cultured with U87 glioblastoma cells expressing GFP (U87-GFP) in EBM, all on a Matrigel substratum to promote tubule formation. After treatment of HBMVECs with DZNep, or transfection with pre-miR-101, EZH2 siRNA, or controls, and subsequent culturing on Matrigel, we analyzed endothelial tubule length and tubule branching. Tubules were visualized by a combination of light and fluorescence microscopy, to visualize the endothelial cells and U87-GFP cells, respectively (Fig. 5A). Up-regulation of miR-101 resulted in a significant decrease in total tubule length and tubule branching (Fig. 5A and B). In addition, treatment with EZH2 siRNA and DZNep also inhibited growth factor- or glioblastoma-induced HBMVEC tubule network formation (Fig. 5A and B). In HUVECs treatment with EZH2 siRNA and pre-miR-101 also led to a significant decrease in tubule length. DZNep treatment did not significantly reduce tubule length in HUVECs, although the quality of the resulting networks seemed less than in the control condition (Fig. S1C and D). These results indicate that EZH2 is involved in endothelial tubule formation.

### Inhibition of EZH2 reduces endothelial migration and invasion *in vitro*

**Figure 6:**
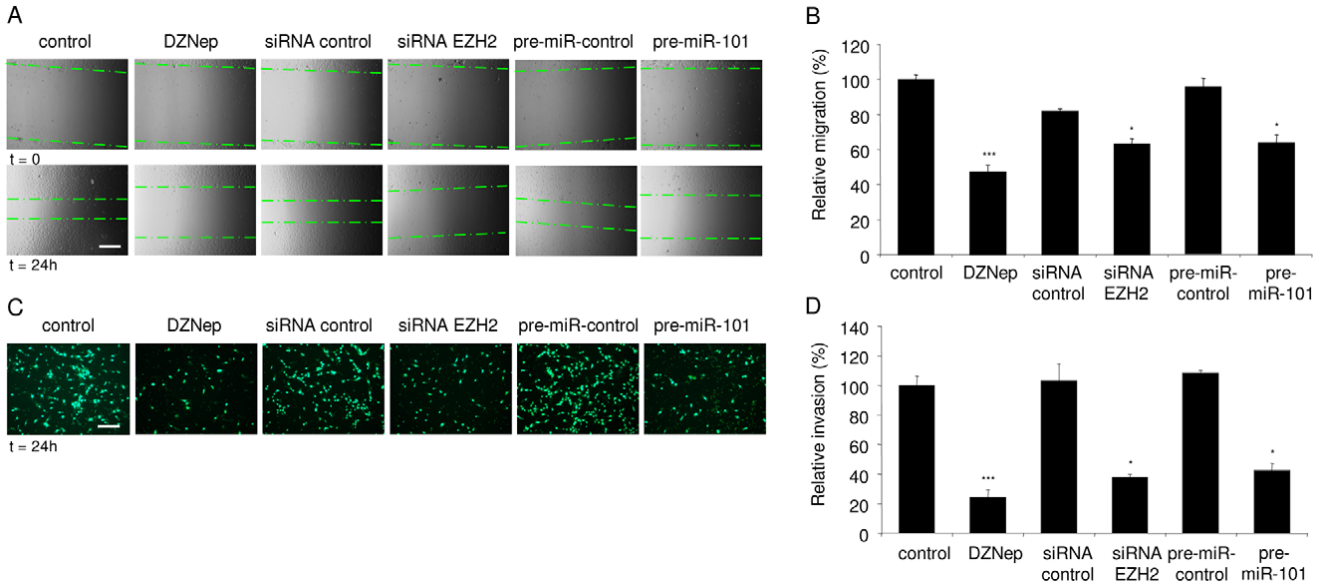
Inhibition of EZH2 reduces endothelial migration and invasion *in vitro*. (A) HBMVEC monolayer cultures were scratched. Images were acquired directly after scratching (t = 0) and 24 h later (t = 24). The migration front is indicated by the dashed lines. Scale bar  = 450 µm. Inhibition of EZH2 in HBMVECs, either by transfection with pre-miR-101, EZH2 siRNA or treatment with DZNep significantly reduced migration as compared to control. (B) Quantitation of endothelial cell migration into the scratched area using ImageJ software. (C and D) HBMVECs were transfected with pre-miR-101, EZH2 siRNA, non-related control molecules, or treated with DZNep, incubated on a Transwell system and subsequently analyzed for invasion capability. EZH2 inhibition, either through pre-miR-101, EZH2 siRNA or DZNep, significantly decreased invasion as shown by Hoechst staining. Scale bar  = 225 µm. (n = 3) Error bars indicate s.d. *p<0.05, ***p<0.001, t test.

Endothelial migration and invasion are two other essential steps in the tumor vascularization process. In order to determine the effect of EZH2 inhibition on growth factor-induced endothelial cell migration, scratch assays were performed. HBMVEC cells were treated with either pre-miR-101, EZH2 siRNA, or DZNep, and cultured in EGM. To promote migration a scratch was made in the confluent HBMVEC monolayer and the migration distance of the HBMVECs into the scratched area was measured and quantitated 24 h later (Fig. 6A and B). Inhibition of EZH2 by pre-miR-101, EZH2 siRNA, or DZNep, resulted in a significant decrease in HBMVEC migration (Fig. 6A and B). We did not detect significant differences in HBMVEC proliferation over the same period, only after a 72 h period HBMVECs proliferation was negatively affected (Fig. S2A and B). To further evaluate the effects of miR-101/EZH2 modulation on endothelial invasion, a Boyden chamber Transwell assay was used. HBMVECs transfected with pre-miR-101 were seeded on the porous membrane of the Transwell insert and the ability of these cells to invade into the lower compartment was measured after 24 h. HBMVECs transfected with pre-miR-101 showed a 60% decrease in their ability to invade compared to control transfected cells, as visualized by Hoechst staining and quantification of the number of invaded cells (Fig. 6C and D). Similar results were observed after treatment with the EZH2 inhibitor DZNep or EZH2 knock down by siRNA (Fig. 6C and D). These results support a functional role for the miR-101/EZH2-axis in the angiogenesis process, where deregulation of EZH2, through EZH2 knockdown or by using the EZH2 small molecule inhibitor DZNep, mimics the anti-angiogenic effects observed after up-regulating miR-101 levels.

### EZH2 inhibitor DZNep reduces glioblastoma-induced angiogenesis *in vivo*

**Figure 7:**
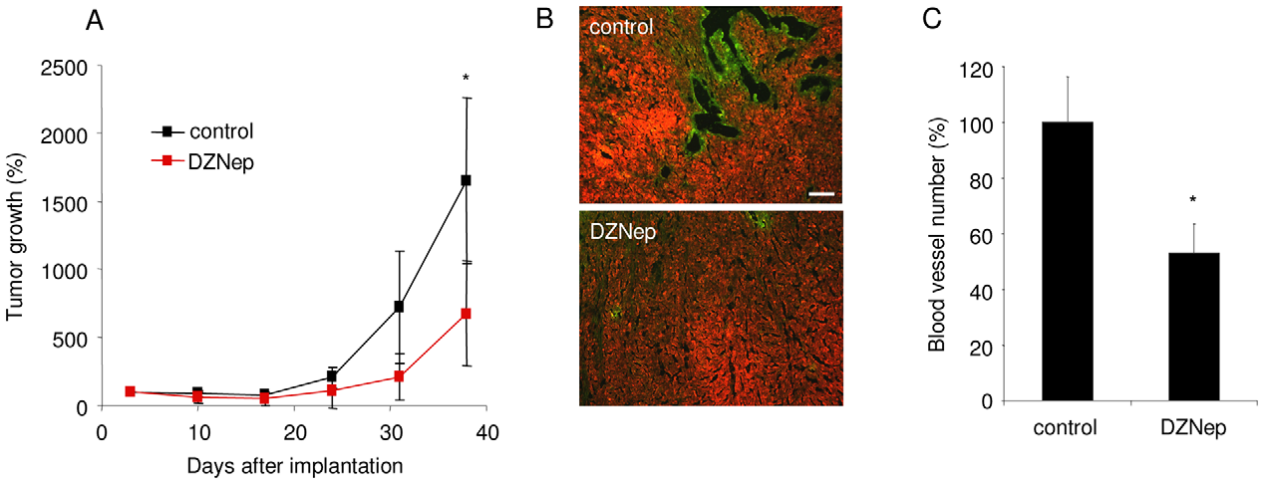
EZH2 inhibitor DZNep reduced glioblastoma-induced angiogenesis *in vivo*. (A) A total of 1×10^6^ U87-Fluc-mCherry cells were implanted s.c. and imaged with a CCD camera 3 days later. At days 3, 5 and 7 after implantation followed by weekly injection, one set of mice was injected i.v. with DZNep and another set with PBS. Tumor growth was monitored by *in vivo* bioluminescence imaging. (B) Lectin-FITC was injected i.v. prior to sacrificing the mice and was used to determine the tumor blood vessels. (C) Quantification of (B). (n = 5) Error bars indicate s.d. *p<0.05, ***p<0.001, t test.

To study the anti-angiogenic effect elicited by EZH2 inhibition *in vivo*, we used a subcutaneous glioblastoma mouse model and the EZH2 inhibitor DZNep. U87 glioblastoma cells, stably expressing the bioluminescence reporter Fluc and the fluorescence reporter mCherry, were implanted in the flank of nude mice (N = 10). Intravenous (i.v.) injections of the EZH2 histone methyltransferase inhibitor DZNep (0.07 mg/kg) were performed on day 3, 5 and 7 after implantation of the cells followed by weekly injection. Tumor growth was monitored using *in vivo* Fluc bioluminescence imaging after injection of its substrate D-Luciferin and acquiring photon counts using a CCD camera. Tumor volume in control mice increased exponentially over time, while tumors in mice treated with DZNep showed reduced growth (Fig. 7A). Ki67 staining of tumor sections revealed areas with proliferating cells in both DZNep treated and control mice (Fig. S3A). Prior to sacrifice, the mice were injected with Lectin-FITC to mark blood vessels. Upon immunofluorescence analysis of tumor tissue slices we noticed that DZNep treatment significantly reduced both the number and the size of the tumor blood vessels, as illustrated by Lectin-FITC blood vessels of mCherry-expressing glioblastoma sections (Fig. 7B and Fig. 7C). This indicates that the EZH2 inhibitor DZNep can inhibit angiogenesis *in vivo*, and suggests a possible therapeutic potential for EZH2 inhibitors in the many diseases in which aberrant angiogenesis plays a critical role.

## Discussion

We have analyzed miR-101 expression in primary HBMVECs. We showed that VEGF down-regulates the expression of miR-101 resulting in increased EZH2 protein expression and causing a pro-angiogenic response in the endothelial cells. Further, we show that this response can be overcome by pharmacological targeting of EZH2 *in vitro* and *in vivo* using the histone methyltransferase inhibitor DZNep. Altogether these results support a role for diminished miR-101-mediated suppression of EZH2 in promoting neovascularization.

Formation of new blood vessels requires endothelial cells to undergo a balanced angiogenic switch. This involves increased expression and secretion of growth factors like VEGF. VEGF modulates destabilization, proliferation, invasion, and sprouting of vessels, thereby orchestrating the formation of neovasculature via signaling through its receptors VEGFR1 and VEGFR2. The proper execution of these processes relies on a concerted action of multiple proteins. It is now clear that miRNAs can orchestrate specific biological processes through post-transcriptional regulation of gene expression. It has also been shown that specific miRNAs are responsible for regulation of endothelial gene expression during angiogenesis [36]–[38]. To determine the miRNA signature of endothelial cells, Poliseno and colleagues generated miRNA expression profiles of HUVECs. They identified 27 highly expressed miRNAs, 15 of which were predicted to regulate the expression of receptors for angiogenic factors [8]. We recently showed that the miRNA expression profile of HBMVECs was modulated upon exposure of these brain endothelial cells to glioblastoma cells [11]. Further, it was shown that endothelial miRNAs can be deregulated by exposure to VEGF [11], [38], [39]. Here we show that U87 conditioned medium and VEGF cause down-regulation of miR-101 in HBMVECs, subsequently resulting in up-regulation of the PcG protein EZH2. Interestingly, we were not able to measure significant and reproducible miR-101 down-regulation in HUVECs upon exposure to U87 conditioned medium, in contrast to HBMVECs. These results indicate a discrepancy between miR-101 regulation in HUVECs and HBMVECs in the context of glioma. However, also in HUVECs EZH2 protein levels could be modulated by EZH2 siRNA, pre-miR-101 or DZNep, and this affected tubule formation. In addition to the mechanism of U87cm and VEGF-suppressed miR-101-mediated translational repression of EZH2 that we describe, Lu et al. have recently shown that VEGF can also increase EZH2 promoter activity [35]. Further research is warranted in order to investigate the different modes of EZH2 regulation in different types of endothelial cells and whether these mechanisms are mutual exclusive.

We confirmed that EZH2 translation is suppressed by miR-101. It was recently shown that miR-101 can regulate EZH2 expression in cancer cells and affects cancer cell migration and invasion [26], [28]. Moreover, it was described that EZH2 can be regulated by miR-26 [24] and miR-214 [25]. Of note, we found both miR-26 and miR-214 to be expressed in HBMVECs (data not shown). However, in primary HBMVECs exposed to glioblastoma cells we found miR-26 and miR-214 not to be deregulated [11]. Here, we show that EZH2 up-regulation in angiogenic brain endothelial cells can be caused by reduced suppression by miR-101, although at this point we cannot exclude that miR-26, miR-214, or other miRNAs, also affect translation of EZH2 in angiogenic endothelial cells.

EZH2 promotes cancer cell proliferation *in vitro* and *in vivo* [40]–[43]. This indicates a potential dual role for EZH2 in endothelial cells and in glioma cells. Regarding the effects of EZH2 inhibition *in vivo*, we performed Ki67 staining on tumor slices of mice treated with or without the EZH2 inhibitor DZNep. Although we found a significant reduction in the number of blood vessels in the DZNep treated tumors we also noticed that proliferation of the glioma cells was still evident (Fig. S3), suggesting that the observed reduction in blood vessels under these conditions was - at least partly - caused by the inhibitory effect of DZNep on the blood vessel endothelial cells, paralleling the results of our *in vitro* angiogenesis assays. However, we do not exclude possible partial effects of DZNep on glioma cell proliferation *in vivo* and a consequent reduction in blood vessel number. It should be noted that our *in vivo* results were obtained in an immune-compromised setting, it would be also of interest to study the effects of EZH2 inhibition in a syngeneic immune-competent model.

Inhibition of up-regulated EZH2 in angiogenic endothelial cells caused reduced migration, invasion, and tubule formation *in vitro* and diminished blood vessel formation in glioblastoma tumors *in vivo*. Here, we describe a new role for the histone methyltransferase EZH2 to stimulate a pro-angiogenic phenotype of endothelial cells. Inhibition of EZH2 by pre-miR-101, EZH2 siRNA, or the small molecule inhibitor DZNep shifted the pro-angiogenic status to a more stationary phenotype. Previously, expression profiling of EZH2-regulated genes indicated that EZH2 can act as a bona fide oncogene that stimulates an ‘active’ cell status by trimethylating lysine 27 of histone H3 [26], [40]–[46]. In addition, it was described that EZH2 suppression, besides loss of H3K27me3, resulted in increased H3 acetylation, and that EZH2 modulation can affect the regulation of genes involved in endothelial differentiation [22], [23]. Histone deacetylase proteins were also described to affect angiogenesis [12], [13] and to revert EZH2-mediated gene silencing [40], [47]. In endothelial cells it remains to be investigated how histone modifications can affect the balance towards a more pro-angiogenic cellular state. Here we provide evidence of a functional link between growth factors, miRNAs, and the histone methyltransferase EZH2 in the angiogenesis process.

## Materials and Methods

### Cells

Human brain microvascular endothelial cells (HBMVECs; Cell Systems ACBRI-376) were cultured in EGM medium (Lonza). 293T cells and U87 cells (U-87 MG; ATCC) were cultured in DMEM containing 10% FBS and antibiotics. U87-Fluc-mCherry and U87-GFP cells were produced by stably transducing U87 cells with CMV-controlled expression cassettes using lentiviral vectors.

### RNA isolation

Total RNA was isolated from HBMVECs, primary endothelial cells isolated from normal brain and gliomas. RNA isolation was carried out by adding 600 µl lysis buffer from the miRVANA miRNA isolation kit (Ambion) to the cell pellets. The isolation of endothelial cells from normal human brain and human gliomas is described elsewhere [48].

### miRNA modulation

50 nM of pre-miR-101 (Ambion) or pre-miR-control (Ambion) oligonucleotides were transfected into HBMVECs using Lipofectamin2000 (Invitrogen), according the manufacturer's protocol. For the inhibition of EZH2, 50 nM of EZH2 siRNA (Qiagen) was transfected into HBMVECs, siRNA-AF or non-targeting oligonucleotides (Qiagen) were used as controls. After 5 h the transfection medium was replaced by EGM until further analysis.

### Quantitive RT-PCR

Quantitive RT-PCR (qRT-PCR) analysis was used to determine the relative expression levels of miR-101, miR-186, EZH2, and GAPDH mRNA in HBMVECs. Total RNA was isolated using the miRVANA miRNA isolation kit. Equal amounts of RNA were converted into cDNA using miR-101, miR-186, EZH2, and GAPDH RT primers (Applied Biosystems and Qiagen, according to the manufacturer's protocol). Subsequently, quantitive PCR was performed using primers and materials from Applied Biosystems. The Ct values were used to calculate the relative fold difference in miRNA or mRNA levels. All experiments were performed using biological triplicates and experimental duplicates. The data were normalized to miR-186 and/or GAPDH expression levels. Primers used were:

human EZH2 5′-CCTGAAGTATGTCGGCATCGAAAGAG-3′ (forward)

5′-TGCAAAAATTCACTGGTACAAAACACT-3′ (reverse)

human GAPDH 5′-GTCGGAGTCAACGGATT-3′ (forward)

5′-AAGCTTCCCGTTCTCAG-3′ (reverse)

### Luciferase miRNA reporter assay

293T cells were transfected with firefly luciferase reporter vectors containing EZH2 wt or control 3′-UTR [24]. At 4 h after transfection, the cells were transfected again with 50 nM miR-101 precursor (Ambion) or controls (Ambion). The cells were lysed after 24 h, and luciferase activity was measured using a luminometer. A total of 100 ng of pCSCW-Gluc [49] was co-transfected and used to normalize the firefly luciferase values expressed from the EZH2 3′UTR report constructs.

### Western blot analysis

72 h after transfection with 50 nM of oligonucleotides or treatment with 5 µM DZNep, or after 24 h of culturing in either EBM, EGM or EBM conditioned culture medium derived from U87 glioblastoma cells, HBMVECs were washed, trypsin treated and harvested, centrifuged (4 min at 1200 rpm) and washed with ice-cold PBS for two times and subsequently resuspended in RIPA buffer including protease inhibitor. After 1 h cells were centrifuged for 10 min at 10,000 rpm and 4°C. Supernatant was mixed with Laemmli buffer (Bio-RAD) including β-mercaptoethanol and heated at 97°C. 30 µg of protein was loaded on a 10% SDS-polyacrylamide gel and separated for 1 h at 100 V on an electrophoresis system (Bio-Rad). Next, proteins were transferred to PVDF membranes overnight at 30 V and 4°C by means of blot buffer (TRIS/glycine/methanol). After incubating the PVDF membranes with PBS for 15 min they were blocked with 5% milk in PBS-T for 1 h. Membranes were incubated for 1 h with purified mouse anti-EZH2 mAb (BD biosciences) at 1∶1,000 dilution in blocking solution (1% milk in PBS-T) and mouse anti-Actin (Millipore, MAB1501R) at 1∶50,000 dilution or for 24 h with rabbit anti-H3K27Me3 (1∶1,000; Upstate Biotechnology). Membranes were washed with PBS-T four times for 15 min. Subsequently, membranes were incubated for 1 h with horseradish peroxidise (HRP) anti-mouse IgG (DAKO) at 1∶3,000 dilution in 1% block buffer to detect primary anti-EZH2 antibody and anti-Actin antibody or HRP anti-rabbit IgG (DAKO) at 1∶3,000 dilution to detect anti-H3K27Me3 antibody. Membranes were washed again with PBS-T four times for 15 min. ECL detection solution (GE healthcare) was used to detect protein levels. Levels were visualized on X-ray film (GE healthcare) and quantified using ImageJ software (NIH Image). Actin intensities were used to normalise EZH2 levels. Normalised EZH2 intensities were used to calculate the relative expression level of EZH2.

### Immunohistochemical staining

Paraffin sections of human glioblastoma tissue and neocortex were stained with monoclonal mouse anti-EZH2 (BD biosciences) at 1∶300 diluted in antibody solution (Immunologic) for 1 h at room temperature. Sections were washed in PBS three times and incubated with secondary antibody (EnvisionHRP) for 30 min at room temperature. After washing in PBS for three times, positive reactions were visualized by incubating the sections with stable 3,3-diaminobenzidine for 10 min. In case of double staining, the sections were subsequently incubated with anti-CD31 (DAKO) at 1∶50 diluted in antibody diluent for 1 h at room temperature, washed three times in PBS, incubated with a biotinylated rabbit anti-mouse secondary antibody (DAKO) at 1∶100 diluted in antibody diluent for 30 min at room temperature, washed in PBS three times and finally incubated with a streptavidin alcalic phosphatase complex (Roche) at 1∶100 diluted in antibody diluent for 1 h at room temperature. Liquid Permanent Red chromogen was used to visualize positive reactions. After washing in distilled water, the sections were counterstained with hematoxylin for 1 min and analyzed using microscopy.

### Microarray expression analysis

mRNA expression arrays were performed at the VUMC array core facility. HBMVECs cultured in EBM + VEGF (10 ng/ml) were compared to HBMVECs cultured in EBM only and HBMVECs transfected with EZH2 siRNA and cultured in EBM + VEGF (10 ng/ml) were compared to HBMVECs transfected with control siRNA and cultured in EBM + VEGF (10 ng/ml). Total RNA was isolated after 24 h of culturing using TRIzol reagent (Invitrogen) according to the manufacturer's protocol. RNA was quantified using a Nanodrop ND 1000 spectrophotometer (Nanodrop Technologies). RNA integrity was assessed using the Agilent 2100 Bioanalyzer (Agilent) and RNA 6000 Nano LabChip kit (5065–4476). RNA integrity numbers (RIN) of >9.0 were considered as good quality RNA. RNA samples were labeled using the Agilent Low RNA Input Linear Amplification Kit Plus (5188–5340) according to the manufacturer's protocol. Briefly, 500 ng of total RNA was amplified and reverse transcribed to cDNA using T7-polymerase and subsequently labeled with Cy3 or Cy5. Dye incorporation was measured using a Nanodrop ND-1000 spectrophotometer. Subsequently, cRNA was hybridized using the Agilent Gene Expression Hybridization Kit (5188–5242), according to the manufacturer's protocol. Briefly, 825 ng of Cy3 labeled cRNA was mixed with 825 ng of Cy5 labeled cRNA, fragmented for 30 min at 60°C in the dark and hybridized on an Agilent Hybridization Chamber Gasket Slide (G2534-60011) in a rotation oven at 65°C for 17 h. Slides scanned using an Agilent Microarray Scanner (G2565BA). Image analysis was performed using feature extraction software version 9.5 (Agilent Technologies). The Agilent GE2-v5_95 protocol was applied using default settings.

Data pre-processing and analysis was performed using the R-Bioconductor package Limma [50]. A robust Edwards background correction was applied. Within-array and between-array normalization was performed using loess and scale standardization. For EZH2 siRNA transfected versus control siRNA transfected cells the highest 5 percentile of differentially expressed log2 expression ratios [range log2 expression ratio: 0.32–1.76] were selected as potential target genes for EZH2. For EBM + VEGF (10 ng/ml) versus EBM cultured cells the lowest 5% percentile differentially expressed log2 expression ratios [range log2 expression ratio: −4.20–−0.48] were selected as potential target genes for VEGF induced EZH2 gene silencing. Subsequently, an overlap set of genes both induced by EZH2 knock down and reduced by VEGF was established as a VEGF-induced-EZH2-target gene set.

### *In vitro* Matrigel assay

A Matrigel (BD biosciences) assay was performed to assess endothelial tube formation *in vitro*. 48-well Plates were coated with 75 µl of Matrigel per well and incubated at 37°C for 15 min. 72 h after transfection with 50 nM of oligonucleotides or treatment with 5 µM DZNep, HBMVECs were harvested and suspended in either EBM, EBM supplemented with U87-GFP cells or EGM. Per well, 60,000 HBMVECs were plated and maintained at 37°C. At least 3 random pictures were taken per well using a digital camera system (Leica), 16 h after plating. The images were analyzed for total tube length and number of branching points using the software program ImageJ. Experiments were performed in triplicate, repeated at least once and judged in a double blind fashion by two observers. Statistical analysis of the results of 3 experiments was performed by Student t-test.

### Wound healing assay

HBMVECs were cultured in EGM in 24-wells plates and were transfected with 50 nM of oligonucleotides or treated with DZNep (5 µM). At 72 h after transfection an artificial wound was created using a pipette tip after which the cells were further incubated. To analyse cell migration into the wound, pictures were taken at 0 h and 24 h using a digital camera system coupled to a microscope. ImageJ was used to determine the migration distance (in µm) as the reduction of the width of the open area. Statistical analysis of the results of 3 experiments was performed by Student's t-test.

### Cell invasion assay

HBMVEC migratory function was analysed using a Boyden chamber assay. A 24-well Transwell system (Corning) was used, with each well containing a permeable Transwell insert containing a 6.5 mm polycarbonate membrane with 8 µm pores. The inserts were coated with 4x diluted basement membrane extract (Trevigen) and incubated overnight in 5% CO2 at 37°C. At 72 h after transfection with 50 nM of oligonucleotides or treatment with 5 µM DZNep cells were starved for 24 h and harvested in serum free medium. Per insert 25,000 HBMVECs were subsequently placed on the membrane. The inserts were immersed in a 24-well plate that was filled with EGM growth medium culture media. After incubation for 24 h, the membrane was washed briefly with PBS. The upper side of the membrane was then wiped gently with a cotton ball. The membrane was then fixed in 4% formaldehyde and stained with Hoechst. The magnitude of HBMVEC migration was evaluated by counting the migrated cells in 3 random high-power (5x) microscope fields. Statistical analysis of the results of 3 experiments was performed by Student's t-test.

### *In vivo* glioblastoma angiogenesis model

All animal studies were approved by the Massachusetts General Hospital Review Board. Protocol approved by the MGH Subcommittee on Research Animal Care (SRAC) - OLAW assurance number A3596-01. Nude mice were anesthetised with i.p. injection of xylazine (5 mg/kg) and ketamine (100 mg/kg). 50 µl containing 1×106 U87-Fluc-mCherry cells was pre-mixed with an equal volume of matrigel (BD biosciences) and implanted in the flanks of nude mice. DZNep was administered intravenously to tumor-bearing mice at a dose of 0.07 mg/kg diluted in 100 µl PBS at day 3, 5 and 7 after tumor implantation, followed by weekly injection. Mice were anesthetized as above and Fluc imaging was performed 10 min after intravenous injection of 150 µl beetle D-luciferin (4 mg/kg body weight) (Xenogen), and recording photon counts over 5 min using a cooled CCD camera with no illumination. Dim polychromatic illumination was used to take a light image of the animal. Visualization was performed using CMIR-Image, a program developed by the Center for Molecular Imaging Research using image display and analysis suite developed in IDL (Research Systems Inc., Boulder, CO). An intensity contour procedure to identify bioluminescence signals with intensities significantly greater than the background was used to define regions of interest. The mean, standard deviation and sum of photon counts in these regions were calculated as a measurement of Fluc activity. Prior to sacrifice the mice were injected intravenously with 150 µl (1 mg/ml) Lectin-FITC (Vector Laboratories) to mark the blood vessels. Animals were sacrificed by transcardial perfusion with 4% paraformaldehyde in PBS under deep anaesthesia. Tumors were removed, post-fixed in paraformaldehyde and PBS containing 30% sucrose, and sectioned into 10 µm sections that were mounted on slides and evaluated for number of blood vessels.

## Supporting Information

Figure S1 **miR-101 and EZH2 modulation and functionality in HUVECs.** (A) qRT-PCR analysis of miR-101 levels in HUVECs exposed to U87 secreted factors (n = 3). (B) HUVECs protein expression analysis of EZH2 following culturing in either EBM, EGM or EBM derived from U87 glioblastoma cells. Numbers indicate the relative expression of EZH2 compared to cells cultured in EBM. (C) HUVECs protein expression analysis of EZH2 and H3K27me3 following transfection of HUVECs with pre-miR-101, EZH2 siRNA, non-related control molecules or treatment with DZNep. Numbers indicate relative EZH2 protein expression normalized against β-Actin expression. (D) HUVECs were cultured on Matrigel coated plates in EGM. Inhibition of EZH2 in HUVECs, either by transfection with pre-miR-101 or EZH2 siRNA, significantly reduced tubule formation as compared to control cells. Tubule formation was assessed as tubule length. (n = 4) Error bars indicate s.d. *p<0.05, ***p<0.001, t test.
(TIF)

Figure S2 **HBMVEC proliferation.** (A) HBMVEC proliferation over 24 h as measured by WST assay following transfection of HBMVEC with pre-miR-101, EZH2 siRNA, non-related control molecules or treatment with DZNep (n = 5). (B) HBMVEC proliferation over 72 h as measured by WST assay following transfection of HBMVEC with pre-miR-101, EZH2 siRNA, non-related control molecules or treatment with DZNep (n = 5).
(TIF)

Figure S3 ***In vivo*** **proliferation.** (A) Immunohistochemical staining for Ki67 in glioblastoma sections harvested from U87 tumor bearing mice treated with either PBS or DZNep. Scale bar  = 50 µm.
(TIF)

## References

[1] Carmeliet P (2005). Angiogenesis in life, disease and medicine.. Nature.

[2] Folkman J (2007). Angiogenesis: an organizing principle for drug discovery?. Nat Rev Drug Discov.

[3] Jain RK (2005). Normalization of tumor vasculature: an emerging concept in antiangiogenic therapy.. Science.

[4] Folkman J (1995). Angiogenesis in cancer, vascular, rheumatoid and other disease.. Nat Med.

[5] Folkman J (2002). Role of angiogenesis in tumor growth and metastasis.. Semin Oncol.

[6] Hanahan D, Folkman J (1996). Patterns and emerging mechanisms of the angiogenic switch during tumorigenesis.. Cell.

[7] Olsson AK, Dimberg A, Kreuger J, Claesson-Welsh L (2006). VEGF receptor signalling - in control of vascular function.. Nat Rev Mol Cell Biol.

[8] Poliseno L, Tuccoli A, Mariani L, Evangelista M, Citti L (2006). MicroRNAs modulate the angiogenic properties of HUVECs.. Blood.

[9] Suarez Y, Fernandez-Hernando C, Pober JS, Sessa WC (2007). Dicer dependent microRNAs regulate gene expression and functions in human endothelial cells.. Circ Res.

[10] Ambros V (2004). The functions of animal microRNAs.. Nature.

[11] Wurdinger T, Tannous BA, Saydam O, Skog J, Grau S (2008). miR-296 regulates growth factor receptor overexpression in angiogenic endothelial cells.. Cancer Cell.

[12] Rossig L, Li H, Fisslthaler B, Urbich C, Fleming I (2002). Inhibitors of histone deacetylation downregulate the expression of endothelial nitric oxide synthase and compromise endothelial cell function in vasorelaxation and angiogenesis.. Circ Res.

[13] Urbich C, Rossig L, Kaluza D, Potente M, Boeckel JN (2009). HDAC5 is a repressor of angiogenesis and determines the angiogenic gene expression pattern of endothelial cells.. Blood.

[14] Sparmann A, van Lohuizen M (2006). Polycomb silencers control cell fate, development and cancer.. Nat Rev Cancer.

[15] Cao R, Wang L, Wang H, Xia L, Erdjument-Bromage H (2002). Role of histone H3 lysine 27 methylation in Polycomb-group silencing.. Science.

[16] Muller J, Hart CM, Francis NJ, Vargas ML, Sengupta A (2002). Histone methyltransferase activity of a Drosophila Polycomb group repressor complex.. Cell.

[17] Kuzmichev A, Nishioka K, Erdjument-Bromage H, Tempst P, Reinberg D (2002). Histone methyltransferase activity associated with a human multiprotein complex containing the Enhancer of Zeste protein.. Genes Dev.

[18] Cao R, Zhang Y (2004). SUZ12 is required for both the histone methyltransferase activity and the silencing function of the EED-EZH2 complex.. Mol Cell.

[19] Pasini D, Bracken AP, Jensen MR, Lazzerini DE, Helin K (2004). Suz12 is essential for mouse development and for EZH2 histone methyltransferase activity.. EMBO J.

[20] Montgomery ND, Yee D, Chen A, Kalantry S, Chamberlain SJ (2005). The murine polycomb group protein Eed is required for global histone H3 lysine-27 methylation.. Curr Biol.

[21] Lu C, Bonome T, Li Y, Kamat AA, Han LY (2007). Gene alterations identified by expression profiling in tumor-associated endothelial cells from invasive ovarian carcinoma.. Cancer Res.

[22] Burdach S, Plehm S, Unland R, Dirksen U, Borkhardt A (2009). Epigenetic maintenance of stemness and malignancy in peripheral neuroectodermal tumors by EZH2.. Cell Cycle.

[23] Richter GHS, Plehm S, Fasan A, Rossler S, Unland R (2009). EZH2 is a mediator of EWS/FLI1 driven tumor growth and metastasis blocking endothelial and neuro-ectodermal differentiation.. Proc Natl Acad Sci U S A.

[24] Sander S, Bullinger L, Klapproth K, Fiedler K, Kestler HA (2008). MYC stimulates EZH2 expression by repression of its negative regulator miR-26a.. Blood.

[25] Juan AH, Kumar RM, Marx JG, Young RA, Sartorelli V (2009). Mir-214-dependent regulation of the polycomb protein Ezh2 in skeletal muscle and embryonic stem cells.. Mol Cell.

[26] Varambally S, Cao Q, Mani RS, Shankar S, Wang X (2008). Genomic loss of microRNA-101 leads to overexpression of histone methyltransferase EZH2 in cancer.. Science.

[27] Friedman JM, Liang G, Liu CC, Wolff EM, Tsai YC (2009). The Putative Tumor Suppressor microRNA-101 Modulates the Cancer Epigenome by Repressing the Polycomb Group Protein EZH2.. Cancer Res.

[28] Cao P, Deng Z, Wan M, Huang W, Cramer SD (2010). MicroRNA-101 negatively regulates Ezh2 and its expression is modulated by androgen receptor and HIF-1alpha/HIF-1beta.. Mol Cancer.

[29] Gabriely G, Wurdinger T, Kesari S, Esau CC, Burchard J (2008). MicroRNA 21 promotes glioma invasion by targeting matrix metalloproteinase regulators.. Mol Cell Biol.

[30] Griffiths-Jones S, Grocock RJ, van DS, Bateman A, Enright AJ (2006). miRBase: microRNA sequences, targets and gene nomenclature.. Nucleic Acids Res.

[31] Tan J, Yang X, Zhuang L, Jiang X, Chen W (2007). Pharmacologic disruption of Polycomb-repressive complex 2-mediated gene repression selectively induces apoptosis in cancer cells.. Genes Dev.

[32] Glazer RI, Hartman KD, Knode MC, Richard MM, Chiang PK (1986). 3-Deazaneplanocin: a new and potent inhibitor of S-adenosylhomocysteine hydrolase and its effects on human promyelocytic leukemia cell line HL-60.. Biochem Biophys Res Commun.

[33] Louis DN (2006). Molecular pathology of malignant gliomas.. Annu Rev Pathol.

[34] Khodarev NN, Yu J, Labay E, Darga T, Brown CK (2003). Tumour-endothelium interactions in co-culture: coordinated changes of gene expression profiles and phenotypic properties of endothelial cells.. J Cell Sci.

[35] Lu C, Han HD, Mangala LS, li-Fehmi R, Newton CS (2010). Regulation of tumor angiogenesis by EZH2.. Cancer Cell.

[36] Suarez Y, Sessa WC (2009). MicroRNAs as novel regulators of angiogenesis.. Circ Res.

[37] Wurdinger T, Tannous BA (2009). Glioma angiogenesis: Towards novel RNA therapeutics.. Cell Adh Migr.

[38] Heusschen R, van GM, Griffioen AW, Thijssen VL (2010). MicroRNAs in the tumor endothelium: novel controls on the angioregulatory switchboard.. Biochim. Biophys Acta.

[39] Suarez Y, Fernandez-Hernando C, Yu J, Gerber SA, Harrison KD (2008). Dicer-dependent endothelial microRNAs are necessary for postnatal angiogenesis.. Proc Natl Acad Sci U S A.

[40] Varambally S, Dhanasekaran SM, Zhou M, Barrette TR, Kumar-Sinha C (2002). The polycomb group protein EZH2 is involved in progression of prostate cancer.. Nature.

[41] Bracken AP, Pasini D, Capra M, Prosperini E, Colli E (2003). EZH2 is downstream of the pRB-E2F pathway, essential for proliferation and amplified in cancer.. EMBO J.

[42] Kleer CG, Cao Q, Varambally S, Shen R, Ota I (2003). EZH2 is a marker of aggressive breast cancer and promotes neoplastic transformation of breast epithelial cells.. Proc Natl Acad Sci U S A.

[43] Bracken AP, Dietrich N, Pasini D, Hansen KH, Helin K (2006). Genome-wide mapping of Polycomb target genes unravels their roles in cell fate transitions.. Genes Dev.

[44] Croonquist PA, Van Ness B (2005). The polycomb group protein enhancer of zeste homolog 2 (EZH 2) is an oncogene that influences myeloma cell growth and the mutant ras phenotype.. Oncogene.

[45] Gil J, Bernard D, Peters G (2005). Role of polycomb group proteins in stem cell self-renewal and cancer.. DNA Cell Biol.

[46] Raaphorst FM, Meijer CJ, Fieret E, Blokzijl T, Mommers E (2003). Poorly differentiated breast carcinoma is associated with increased expression of the human polycomb group EZH2 gene.. Neoplasia.

[47] van der Vlag J, Otte AP (1999). Transcriptional repression mediated by the human polycomb-group protein EED involves histone deacetylation.. Nat Genet.

[48] Miebach S, Grau S, Hummel V, Rieckmann P, Tonn JC (2006). Isolation and culture of microvascular endothelial cells from gliomas of different WHO grades.. J Neurooncol.

[49] Wurdinger T, Badr C, Pike L, de KR, Weissleder R (2008). A secreted luciferase for ex vivo monitoring of *in vivo* processes.. Nat Methods.

[50] Smyth GK (2004). Linear models and empirical bayes methods for assessing differential expression in microarray experiments.. Stat Appl Genet Mol Biol.

