# Supplementary figures and images for: Down-Regulation of miR-101 in Endothelial Cells Promotes Blood Vessel Formation through Reduced Repression of EZH2
Source: PLoS One. 2011 Jan 28;6(1):e16282. doi: 10.1371/journal.pone.0016282 (PMC3030563; doi:10.1371/journal.pone.0016282)

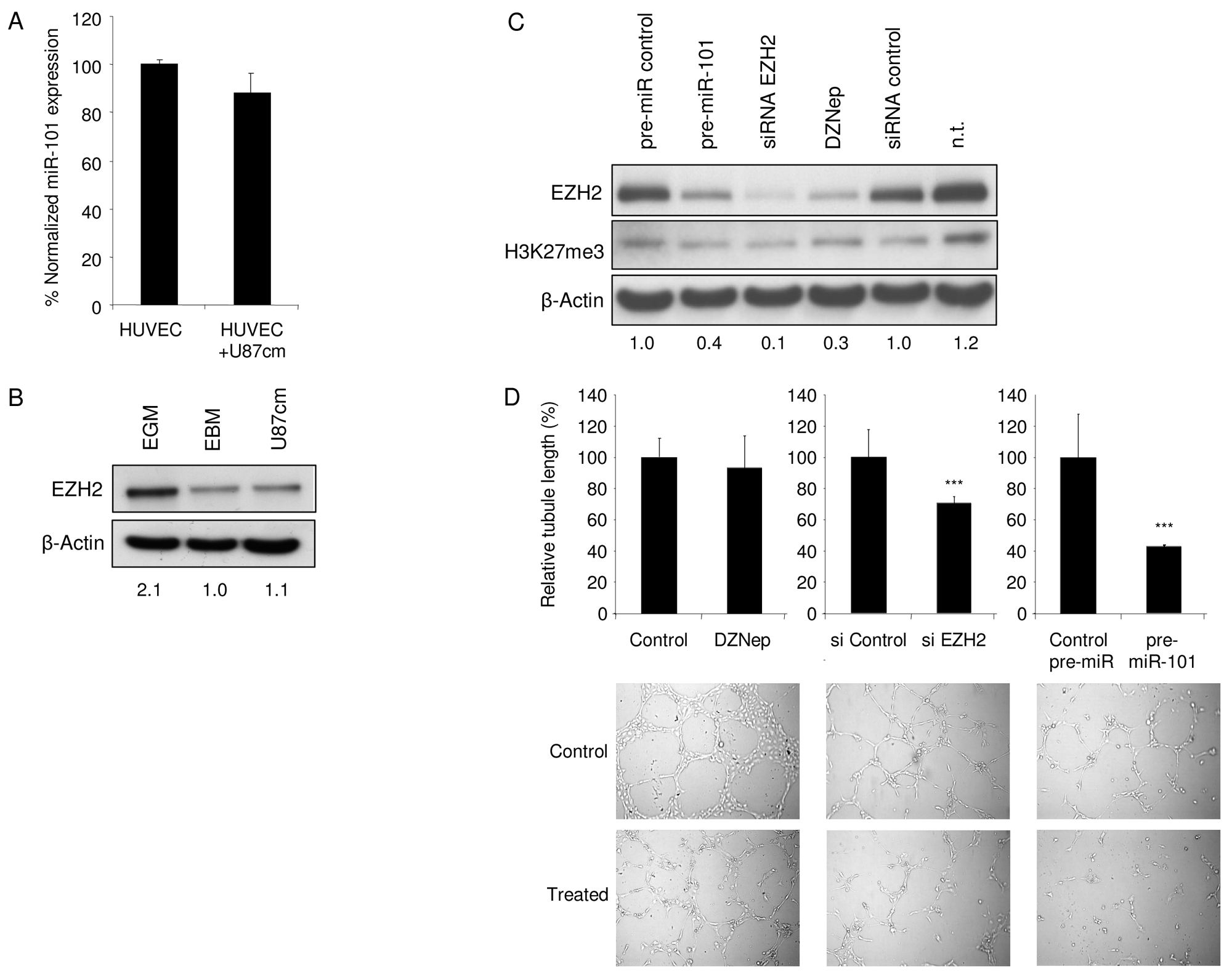

Supplement: Figure S1 — miR-101 and EZH2 modulation and functionality in HUVECs. (A) qRT-PCR analysis of miR-101 levels in HUVECs exposed to U87 secreted factors (n = 3). (B) HUVECs protein expression analysis of EZH2 following culturing in either EBM, EGM or EBM derived from U87 glioblastoma cells. Numbers indicate the relative expression of EZH2 compared to cells cultured in EBM. (C) HUVECs protein expression analysis of EZH2 and H3K27me3 following transfection of HUVECs with pre-miR-101, EZH2 siRNA, non-related control molecules or treatment with DZNep. Numbers indicate relative EZH2 protein expression normalized against β-Actin expression. (D) HUVECs were cultured on Matrigel coated plates in EGM. Inhibition of EZH2 in HUVECs, either by transfection with pre-miR-101 or EZH2 siRNA, significantly reduced tubule formation as compared to control cells. Tubule formation was assessed as tubule length. (n = 4) Error bars indicate s.d. *p<0.05, ***p<0.001, t test. (TIF) [file pone.0016282.s001.tif]

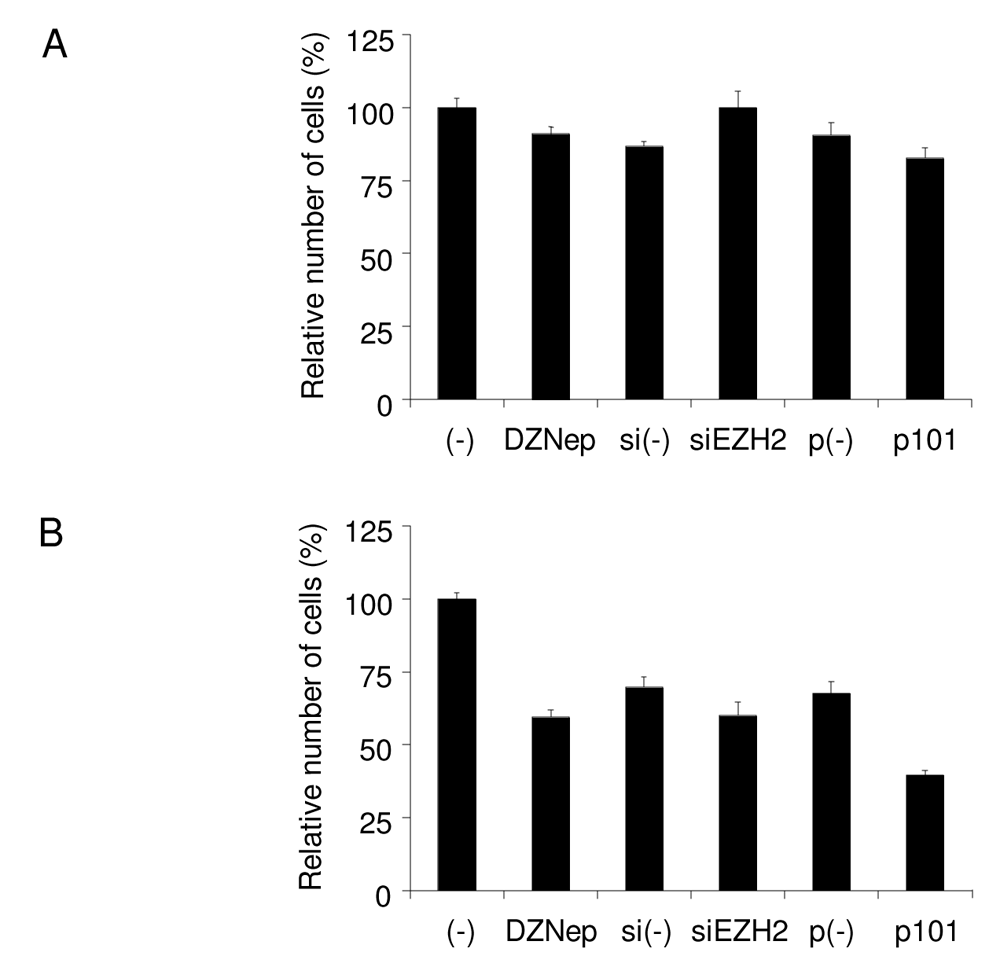

Supplement: Figure S2 — HBMVEC proliferation. (A) HBMVEC proliferation over 24 h as measured by WST assay following transfection of HBMVEC with pre-miR-101, EZH2 siRNA, non-related control molecules or treatment with DZNep (n = 5). (B) HBMVEC proliferation over 72 h as measured by WST assay following transfection of HBMVEC with pre-miR-101, EZH2 siRNA, non-related control molecules or treatment with DZNep (n = 5). (TIF) [file pone.0016282.s002.tif]

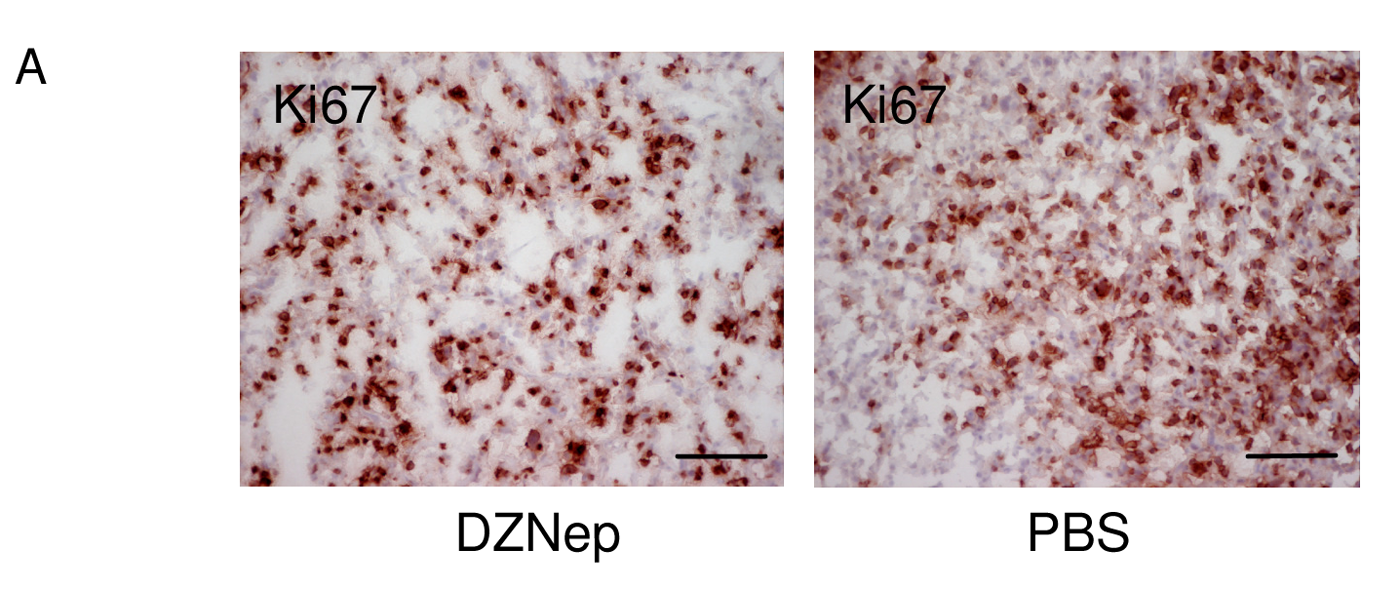

Supplement: Figure S3 — In vivo proliferation. (A) Immunohistochemical staining for Ki67 in glioblastoma sections harvested from U87 tumor bearing mice treated with either PBS or DZNep. Scale bar = 50 µm. (TIF) [file pone.0016282.s003.tif]
